# Nanoparticle-Based Technology Approaches to the Management of Neurological Disorders

**DOI:** 10.3390/ijms21176070

**Published:** 2020-08-23

**Authors:** Tao Ming Sim, Dinesh Tarini, S. Thameem Dheen, Boon Huat Bay, Dinesh Kumar Srinivasan

**Affiliations:** 1 Yong Loo Lin School of Medicine, National University of Singapore, Singapore 117597, Singapore; e0268883@u.nus.edu; 2 Government Kilpauk Medical College, The Tamilnadu Dr MGR Medical University, Chennai, Tamilnadu 600032, India; tarinidinesh@gmail.com; 3 Department of Anatomy, Yong Loo Lin School of Medicine, National University of Singapore, Singapore 117594, Singapore; antstd@nus.edu.sg (S.T.D.); antbaybh@nus.edu.sg (B.H.B.)

**Keywords:** neurological disorders, blood-brain barrier, Alzheimer’s disease, Parkinson’s disease, cerebrovascular diseases, traumatic brain injury, nanoparticles

## Abstract

Neurological disorders are the most devastating and challenging diseases associated with the central nervous system (CNS). The blood-brain barrier (BBB) maintains homeostasis of the brain and contributes towards the maintenance of a very delicate microenvironment, impairing the transport of many therapeutics into the CNS and making the management of common neurological disorders such as Alzheimer’s disease (AD), Parkinson’s disease (PD), cerebrovascular diseases (CVDs) and traumatic brain injury (TBI), exceptionally complicated. Nanoparticle (NP) technology offers a platform for the design of tissue-specific drug carrying systems owing to its versatile and modifiable nature. The prospect of being able to design NPs capable of successfully crossing the BBB, and maintaining a high drug bioavailability in neural parenchyma, has spurred much interest in the field of nanomedicine. NPs, which also come in an array of forms including polymeric NPs, solid lipid nanoparticles (SLNs), quantum dots and liposomes, have the flexibility of being conjugated with various macromolecules, such as surfactants to confer the physical or chemical property desired. These nanodelivery strategies represent potential novel and minimally invasive approaches to the treatment and diagnosis of these neurological disorders. Most of the strategies revolve around the ability of the NPs to cross the BBB via various influx mechanisms, such as adsorptive-mediated transcytosis (AMT) and receptor-mediated transcytosis (RMT), targeting specific biomarkers or lesions unique to that pathological condition, thereby ensuring high tissue-specific targeting and minimizing off-target side effects. In this article, insights into common neurological disorders and challenges of delivering CNS drugs due to the presence of BBB is provided, before an in-depth review of nanoparticle-based theranostic strategies.

## 1. Introduction

**Figure 1:**
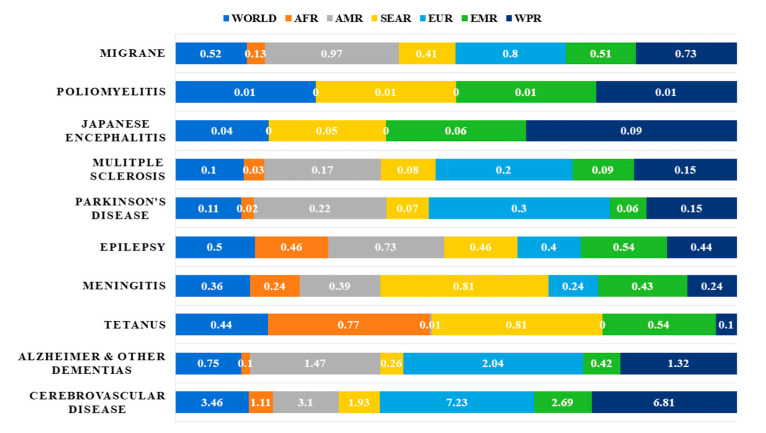
Neurological disorders as percentage of total DALYS by regions as defined by WHO in 2005 [3]. Abbreviations: DALYs: disability-adjusted life years; AFR: Africa Region; AMR: America Region; SEAR: South-East Asia Region; EUR: Europe Region; EMR: Eastern Mediterranean Region; WPR: Western Pacific Region.

The world is currently faced with an ageing population, which has given rise to a trend of increasing incidence of neurological diseases [1]. Neurological disorders account for a significant portion of the global burden of diseases, and it has been estimated that over 1.5 billion people are afflicted with pathogenic neurological diseases [2], such as migraine, poliomyelitis, Parkinson’s Disease (PD) and Alzheimer’s Disease (AD) [3] (Figure 1).

**Figure 2:**
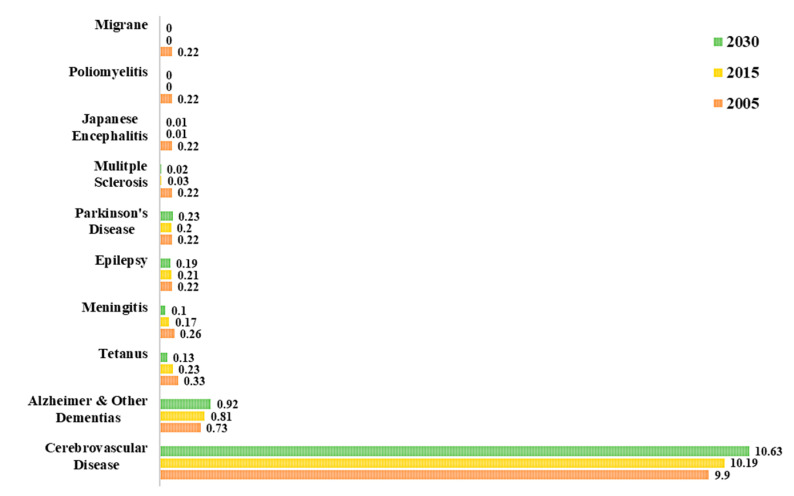
Percentage of total death attributable to neurological disorders in 2005, 2015 and 2030 [3].

In fact, a study on global disease burden in 2016, has highlighted that neurological disorders were the leading cause of global disability-adjusted life-years (DALYs) affecting 276 million people [4]. Statistics from the World Health Organization (WHO) on the percentage of deaths attributed to neurological disorders in the years 2005 and 2015, and a projection for the year 2030 are shown in Figure 2 [3]. Out of all neurological disorders listed, cerebrovascular diseases (CVDs) accounted for about 10% of global deaths in 2015 and represents the neurological disorder with the overall highest mortality. Epilepsy, AD and other dementias, migraine, CVDs and tetanus are some of the neurological disorders that account for the highest percentage of total DALYs. Neurological disorders have been ranked as the second leading cause of deaths (9.0 million) in 2016 [4]

Despite the growing disease burden of neurological disorders, the diagnosis and treatment of brain disorders are still sub-optimal, mainly due to the presence of the blood-brain barrier (BBB), imposing restrictions on the transport of biologically active compounds and drugs [5]. Disorders of the brain and the central nervous system (CNS) can be categorized into different classes, with the three major classes being neurodegenerative, neuroinflammatory and neuro oncological diseases [6]. The most prevalent disorders include Alzheimer’s disease (AD), Parkinson’s disease (PD), traumatic brain injury (TBI) and Tourette syndrome [7].

Many of these disorders do not have effective therapies, not because of a dearth of candidate drugs, but rather, due to the inability of many potential therapeutics to cross the BBB, so as to maintain a high enough bioavailability for significant pharmacological effects in the brain parenchyma [8]. The BBB is a dynamic interface which serves the physiological function of maintaining homeostasis of the brain, by protecting it from invading pathogens and other potentially noxious substances [9]. The presence of circumferential tight junctions between non-fenestrated endothelial cells of the capillaries forming the BBB and efflux transporters, such as P-glycoprotein, are some of the mechanisms which allow the BBB to act as a filter with selective permeability to molecules, including therapeutics [10]. This has profound implications on drug delivery, as almost 100% of large molecule drugs and more than 98% of small molecule drug candidates, are unable to cross the BBB [11].

There has been growing interest in the field of nanomedicine, in providing solutions to the yet unsolved problem of enhancing drug transport across the BBB for imaging, diagnosis, and treatment of neurological disorders [12]. Nanomedicine involves the use of nanoscale materials, such as nanoparticles (NPs), which are biocompatible structures 1–100 nm in size that can be fabricated from a variety of materials including polymers, metals, lipids, and crystals [13]. NPs, owing to their size, behave differently from bulk materials and possess unique properties including the ability to carry a drug payload, high stability in blood, low immunogenicity, high biodegradability and the potential for modification of surface properties [14].

In this review, the pathologies of common neurological disorders namely Alzheimer’s disease (AD), Parkinson’s disease (PD), cerebrovascular diseases (CVDs) and traumatic brain injury (TBI) will be discussed, followed by a description of the BBB which is a major obstacle in the delivery of therapeutics to the brain. Finally, the use of NPs for the potential treatment of neurological disorders will be explored, with emphasis placed on neurodegenerative diseases.

## 2. Neurological Disorders

### 2.1. Neurodegenerative Diseases

Neurodegenerative diseases (with the most common being AD and PD) are disorders characterized by synaptic abnormalities, progressive loss of neurons and the presence of cerebral protein aggregates, resulting in gradual decline in cognition, memory and skilled movements among other symptoms [15]. The exact factors and aetiology of neurodegenerative diseases are still unknown but various risk factors such as environmental pollutants, familial history and genetic variants at several gene loci have been identified [16]. The hereditary cause of neurodegenerative diseases is well-established and there are recent advances exploring the possible use of genetic sequencing of various genetic markers in the diagnosis of neurodegenerative diseases [17].

It is worth noting that numerous studies have suggested that the BBB undergoes pathological alterations, which have been described as a ‘breakdown’ as a consequence of neuroinflammation, resulting in the loss of endothelial tight junction proteins, and thereby an increase in BBB permeability [18]. The sequelae of this phenomenon is that therapeutics would react differently to the brains of patients with neurodegenerative diseases compared to normal brains. Given the dynamic nature of the BBB, the role of pathological alternations to the BBB in affecting drug delivery to the brain is still not well understood, but it should be noted that neurodegenerative brains exhibit different brain pharmacokinetic properties from normal brains [19].

#### 2.1.1. Alzheimer’s Disease (AD)

AD is the most common cause of dementia and up to 80% of all dementia diagnoses can be attributed to AD [20]. It has been identified that the greatest risk factor in the development of AD is aging, with most patients above the age of 65, suffering from AD [21]. AD is a progressive neurodegenerative disease, that most commonly presents as a gradual decline in cognition, with sporadic memory impairments being one of the earliest and most prominent clinical features [22]. As the disease progresses, the decline in cognition causes deficits in speech, visuospatial orientation, and motor function [23]. One of the two primary cardinal neuropathologic lesions in an AD brain, is the presence of abnormal intracellular fibrous inclusions within neuronal perikaryal cytoplasm, referred to as neurofibrillary tangles, which upon histopathological identification can give a definitive diagnosis of AD, though this is rarely done in life and more often done post-mortem [24]. These neurofibrillary tangles are paired helical filaments, formed by aggregates of hyperphosphorylated microtubule-associated protein tau [25].

**Figure 3:**
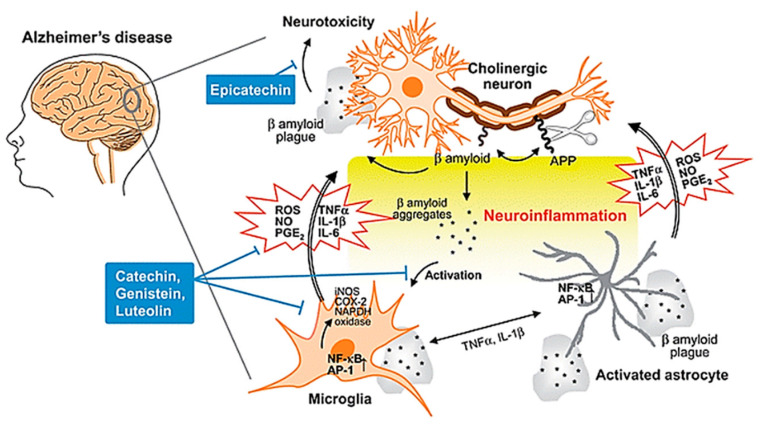
Neuroinflammation in AD. During the development of AD, A*β* peptide is produced by cleavage of APP, aggregates and accumulates as *β* amyloid plaques. Both aggregates and plaques cause neurotoxicity or activation of microglia through up-regulating NF-κB and AP-1 transcription factors, which in turn release ROS and pro-inflammatory cytokines such as NO, PGE2, IL-1, IL-6, and TNF-a that damage cholinergic neuron. These pro-inflammatory cytokines also directly activate astrocytes that also produce cytokines to amplify inflammatory signals and result in neuroinflammation and neurodegeneration. Flavonoids act through avoiding *β* amyloid induced-neuron injury and death, modulation of pro-inflammatory cytokines and ROS production, as well as inhibiting the activation of microglia and astrocyte as neuroprotective mechanisms. Abbreviations: AD: Alzheimer’s disease; APP: amyloid precursor protein; A*β*: amyloid beta; NF-κB: nuclear factor kappa B; AP-1: activator protein 1; ROS: reactive oxygen species; NO: nitric oxide; PGE2: prostaglandin E2; IL-1: interleukin-1; IL-6: interleukin-6; TNF-α: tumor necrosis factor alpha. Reproduced from [30], with permission from Royal Society of Chemistry, 2010.

The other characteristic lesion associated with AD is senile/neuritic plaque [26]. Senile plaques are complex proteinaceous extracellular aggregates of amyloid beta (A*β*) protein, which tend to accumulate in the cortices of the brains of AD patients [27]. In the past few decades, the amyloid cascade hypothesis has been the most well-known theory in accounting for the pathological changes in AD [28]. This theory posits that the central event in AD pathology is the deposition of A*β* plaques which is the causative agent of a cascade of events as depicted in Figure 3, ultimately resulting in neurofibrillary tangles, neuroinflammation, neuronal damage and death, with disrupted neuronal communication, among other pathological presentations of AD [29,30]. However, there have been contradictory results which seem to suggest that the deposition of A*β* plaques may not be the pathogenic cause responsible for the progression of AD, with some studies even suggesting that formation of neurofibrillary tangles precedes the formation of A*β* plaques [31]. Given the conflicting evidence, the true pathogenic role of A*β* in AD pathogenesis is still highly debated and should be subjected to more scientific studies in the future to definitively determine the molecular mechanisms of AD pathogenesis [32].

However, it is well-established that with AD progression, there is a loss of cholinergic neurons, especially in the nucleus basalis of Meynert, which is found in the substantia innominate of the basal forebrain, where degeneration contributes to memory loss in AD patients [33]. Current established treatments of AD are mainly symptomatic in nature and aim to counteract the neurotransmitter acetylcholine (ACh) imbalance in AD patients, which is a direct consequence of the loss of cholinergic neurons [34]. Presently, available treatment options for AD include the use of cholinesterase inhibitors (CIs), with the three CIs approved for prescription being donepezil, galantamine and rivastigmine [35]. These CIs inhibit the enzyme acetylcholinesterase (AChE), which breaks down ACh in the synaptic cleft thereby increasing the bioavailability of ACh, compensating for the loss of cholinergic neurons [36]. However, the efficacy of these drugs is limited, and various side effects have been documented especially at higher doses [37]. Memantine is the 4th drug approved by the Food and Drug Administration (FDA) for use in AD patients, and functions as an N-methyl-D-aspartate (NMDA) receptor antagonist, which blocks the excitatory neurotransmitter glutamate [38]. Memantine, a non-competitive inhibitor of NMDA, is believed to protect neurons from neuronal death and excitotoxicity [39].

Despite treatments options being available for AD, the approved treatments are purely symptomatic and do not serve to prevent further progression of the disease [40]. There have been numerous therapeutic drugs that have shown potential in targeting the A*β* pathway, including tramiprosate, semagacestat and tarenflurbil but have been ultimately unsuccessful in the final phases of clinical trials [41]. Though the reasons for their clinical failure are not clear, low penetration across the BBB has been suggested, especially for the inefficacy of tarenflurbil [42].

#### 2.1.2. Parkinson’s Disease (PD)

PD is another known major neurodegenerative disease globally. PD affects mainly the aged population and it is estimated that the prevalence of the disease is about 1–2 per 1000 people [43]. The onset of PD before the age of 50 is rare and the disease hits a peak prevalence of approximately 4% in older age groups [44]. PD is a complex neurological condition characterized by motor and non-motor deficits such as bradykinesia, resting tremors, rigidity, and postural instability [45]. In the pathogenesis of PD, there is a selective and progressive degeneration of dopaminergic neurons in the substantia nigra pars compacta (SNc), that project into the basal ganglia which causes the aforementioned symptoms of PD [46].

PD is a type of α-synucleinopathy and therefore has a cardinal histopathological feature, which is the presence of abnormal intraneuronal inclusions, composed mainly of α-synuclein called Lewy bodies [47]. It is believed that PD has a strong genetic link, with aberrations in the gene *synuclein alpha (SNCA)* which encodes for α-synuclein, a protein which has been identified as one of the contributory factors for the onset of PD [48]. Aside from the known genetic causes, the pathogenic mechanisms of PD have not been well elucidated, but various factors such as oxidative stress, environmental pollutants and mitochondrial dysfunction, are recognized as predisposing factors of PD [49]. The clinical diagnosis of PD is made via the patient’s symptoms, and a definitive diagnosis can only be confirmed from the presence of Lewy bodies and degeneration of dopaminergic neurons in the SNc at post-mortem [50].

The exact cause of PD is still not well understood, but as a result of advances in genetics, imaging, proteomics and other novel laboratory techniques, exceptional progress has been made to uncover the underlying mechanisms [51]. Regardless of the etiology of the neurodegeneration in PD, the pathogenesis of the loss of dopaminergic neurons is thought to involve either necrosis or apoptosis [52]. Various hypotheses have been proposed to explain the cause of neurodegeneration in PD, with one emerging conjecture being the disruption of intracellular vesicular transport due to microtubular destabilization [53]. Another hypothesis involves mitochondrial dysfunction, which causes neurons to produce inadequate energy to meet metabolic demands, eventually leading to the production of α-synuclein and neuronal damage [54]. There is also growing interest in the role of astrocytes in the pathogenesis of PD, among other neurodegenerative diseases [55].

**Figure 4:**
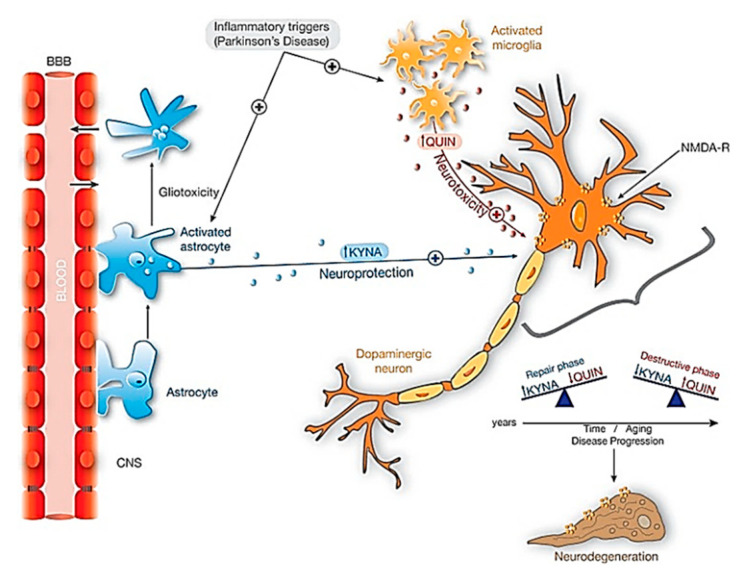
Neuroprotective effects of KYNA against QUIN toxicity in dopaminergic neurons. When inflammatory triggers increased in Parkinsonism, both microglia and astrocytes activate significantly its activity. The activation of astrocytes producing KYNA is essential in this neuroprotection. Additionally, with aging and during disease progression there is an unbalance between KYNA and QUIN: in repair phases the amount of KYNA exceeds the amount of QUIN, and the contrary on the destructive phases and neurodegeneration. Abbreviations: KYNA: kynurenic acid; NMDA-R: NMDA Glutamatergic receptor; QUIN: Quinolinic acid. Reproduced from [58], with permission from Elsevier, 2017.

The kynurenine pathway (KP) is a major degradative pathway of the amino acid tryptophan, which is a pathway implicated in the regulation of immune responses [56]. The KP has been reported to contribute to the neuroinflammation in PD brains and gives rise to various products, one of which is the excitotoxin, quinolinic acid (QUIN), which has been observed to accumulate in the neurons of PD patients, and can lead to neuronal cell death [57]. The molecular cascade of KP summarized in Figure 4, is well documented in various review articles [58].

Similar to the clinical management of AD, the available treatment options for PD are based largely on dopaminergic drugs, which can only alleviate patients’ symptoms, but are unable to slow neurodegeneration and the onset of the disease [59]. Levopoda is a commonly prescribed dopamine agonist and is currently the most potent drug for controlling the symptoms of PD, especially motor deficits related to bradykinesia [60]. Levodopa is commonly administered together with carbidopa. a peripheral dopa decarboxylase inhibitor, which increases the bioavailability of levodopa in the CNS [61]. However, a significant number of patients on levodopa have been found to experience motor fluctuations, dyskinesis and other motor complications, after 5 years of treatment [62].

Despite the current availability of symptomatic treatment options which are also associated with a plethora of adverse side effects, there is still much room for the development of novel treatment approaches such as stem-cell derived grafts and viral gene therapies [63].

### 2.2. Cerebrovascular Diseases (CVDs)

CVDs refer to a group of disorders affecting the vasculature of the brain. This group of diseases involve disorders in which a region of the brain is transiently or permanently affected by ischaemia or bleeding [64]. While there are numerous CVDs such as carotid stenosis, vertebral stenosis, aneurysms and aortic dissections, perhaps a condition of particular clinical interest is stroke, which is the second leading cause of death worldwide and a major cause of disability [65]. There are two broad categories of stroke, namely ischemic stroke involving cerebral vessel occlusions, which accounts for about 87% of all stroke cases in the United States (US), and haemorrhagic stroke involving intracerebral haemorrhage (ICH) accounting for the rest of the cases [66]. Regardless of the aetiology, stroke results in the acute onset of neurological deficits due to a focal CNS injury secondary to a vascular cause [67].

**Figure 5:**
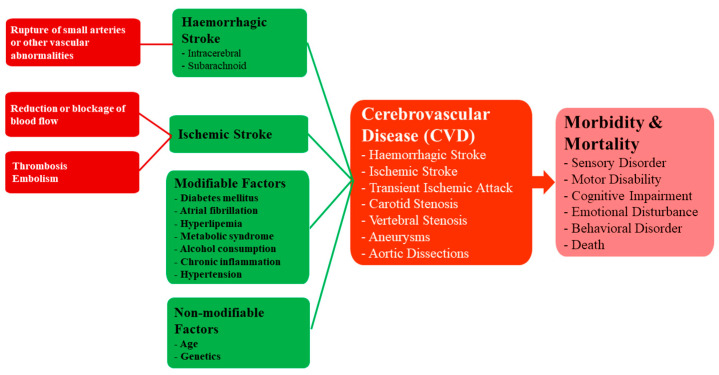
A framework for understanding the causes and consequences of CVD.

Ischaemic stroke is caused by a reduction or blockage of blood flow and has a multitude of causes, including thrombosis and embolism. On the other hand, haemorrhagic stroke is typically caused by the rupture of small penetrating arteries secondary to a chronic hypertensive state or other vascular abnormalities [68]. The risk factors for both ischaemic and haemorrhagic stroke are similar and include modifiable factors such as diabetes mellitus, atrial fibrillation, hyperlipemia, metabolic syndrome, alcohol consumption, chronic inflammation and hypertension, and non-modifiable factors such as age and genetics [69]. A flowchart showing the causes and consequences of CVD Figure 5.

The standard management of acute ischaemic stroke is to focus on the resolution of the thrombus or embolus causing the ischaemia, followed by attempts to revascularize and perfuse the affected brain parenchyma [70]. Intravenous thrombolytic agents, such as recombinant tissue plasminogen activator, comprise the standard first line treatment options for ischaemic stroke caused by an underlying thrombus [71]. In the chronic management of ischaemic stroke, anticoagulants and antiplatelets which interfere with the natural process of haemostasis, are prescribed to prevent recurrent cerebrovascular events from occurring [72].

The onset of haemorrhagic stroke is often sudden and acute; thus, the primary goal of management before the patient reaches the hospital is to provide airway and cardiovascular support, as well as a meticulous recording of the medical history and current medications of the patient, which may aid the physician in adopting the right clinical treatment [73]. The primary focus of clinical management is to provide haemostatic treatment, depending on the cause of the haemorrhage. For example, for warfarin-associated coagulopathies, administration of fresh frozen plasma and prothrombin complex concentrates can be considered and for heparin-associated coagulopathies, administration of protamine sulphate is a common approach [73].

Stroke is a disorder that brings with it many debilitating motor consequences, thus despite the availability of current treatment and diagnostic options, there is a need for further innovation. It is estimated that the typical patient loses 1.9 million neurons with each passing minute that the stroke is untreated [74]. In fact, it is hypothesized that following an episode of stroke where there is a delay in receiving treatment, the number of neurons lost is equivalent to the number lost in 36 years of normal ageing [74]. The time-dependent nature of stroke and its correlated prognosis for the patient are what spur the constant search for more time efficient alternatives.

### 2.3. Traumatic Brain Injury (TBI)

TBI is sometimes referred to as the ‘silent epidemic’, with an estimated 69 million individuals per year suffering from TBI of all causes, contributing to global disability and death more than any other traumatic insult [75]. Survivors of TBI often live with debilitating disabilities, imposing a major economic burden on both the patient and the healthcare system. In fact, the economic repercussion of TBI in the US was estimated to be USD 76.5 billion in 2010 alone [76]. Moreover, TBI was responsible for about 2.5 million emergency department visits, 282,000 cases of hospitalization and 56,000 deaths in the US in 2013 [77].

TBI is most prevalent among the very young (0–4 years of age), young adults (15–24 years of age) and the elderly (>65 years of age) [78]. The two most common causes of TBI are falls and motor vehicle accidents [79]. Aside from possible morbidity, TBI is also known to cause significant motor and cognitive deficits in surviving patients, contributing to chronic functional impairment, thereby adversely affecting quality of life [80]. Possible motor and cognitive sequelae of TBI, include tandem gait impairment and deficits in attention, memory, communication, and intellectual ability [81,82]. TBI is caused by a violent collision or rapid acceleration or deceleration of the brain which leads to altered mental function [83]. In the clinical management of TBI, several factors must be considered, such as primary and secondary brain injuries, which are the two categories that TBI-related brain injuries are classified [84]. Primary injuries are typically defined by direct mechanical force, such as the rapid deceleration of the brain within the cranium, blast waves or penetrating injuries and occur at the time of the trauma [84]. The insult from the primary brain injuries leads to a cascade of molecular events which cause secondary brain injuries that occur over time after the initial incidence of trauma. Possible secondary brain injuries include ionic disturbances [85], neuroinflammation [86], neuronal apoptosis [87], mitochondrial dysfunction [88] and progressive neurodegeneration [89].

Since the primary injury occurs at the time of trauma, it cannot be mended, and thus therapeutic interventions should focus on the management of secondary damage [90]. Current clinical management of TBI primarily involves intensive care management, such as monitoring of haemodynamic and ventilatory functions, thromboembolism prophylaxis, and in particular, the optimization of cerebral oxygenation and cerebral perfusion pressure [91]. However, to date, there is no effective neuroprotective or curative treatment for TBI. In other words, there is a paucity of effective therapy in preventing the increased risk of neurodegeneration and other neurological complications of TBI [92]. It is interesting to note that over the past 3 decades, considerable efforts have been made towards the discovery of novel drugs and therapeutic options for the acute treatment of TBI. Although over 20 late phase II or phase III clinical trials for moderate or severe TBI have been conducted, unfortunately none of the therapies investigated were found to confer overall benefit across the patients involved in the studies [93]. The failure of past clinical trials has been postulated to stem from poor delivery and retention of therapeutics in the brain, off-target toxicity, and a lack of understanding of the pathophysiology of TBI [93].

## 3. Blood Brain Barrier (BBB)—A Challenge for CNS Drug Delivery

**Figure 6:**
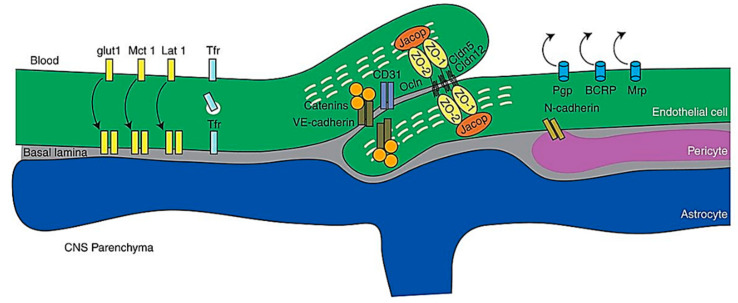
Schematic representation of molecules of the BBB. Abbreviations: CNS, central nervous system; VEcad, VE cadherin. Reproduced from [97], with permission from Cold Spring Harbor, 2017.

The BBB refers to the structure that separates the peripheral circulation from the CNS, where its structural integrity, physiological functions, and molecular mechanisms, integrate harmoniously to control the movement of molecules between the brain and the blood [94]. The property of the BBB as a selectively permeable filter is conferred by the endothelial junctional complexes of tight junctions and adherens junctions in the endothelial lining of the BBB—the tight junctions function to seal the inter-endothelial cleft, thereby forming a continuous blood vessel lumen, while the adherens junctions have been found to mediate endothelial cell–cell contact [95,96]. Other than endothelial cells, the BBB is also comprised of other cell types including mural cells which are vascular smooth muscle cells, astrocytes and pericytes [97] Figure 6.

The molecular mechanisms of transport of substances across the BBB, include efflux and influx mechanisms. It is worth noting that small water-soluble molecules can simply diffuse through the tight junctions of the BBB but not to an appreciable extent [9]. Receptor-mediated transcytosis (RMT) is one of the influx mechanisms of the BBB, which allows for selective uptake of various macromolecules that are too large for simple diffusion [98]. Endothelial cells lining the BBB possess receptors for certain ligands such as plasma proteins, growth factors and other macromolecules. When these receptors bind to the corresponding ligand, the cell membrane invaginates to form intracellular coated vesicles which are subject to processing, allowing for recycling of the receptor and the dissociation of the ligand, before subsequent release at the basolateral surface of the endothelial cell [99]. Macromolecules such as insulin [100], iron [101], and leptin [102] have been shown to cross the BBB via RMT.

Adsorptive-mediated transcytosis (AMT) is another important influx mechanism of the BBB. The conception of AMT came about through observations that polycationic peptides, such as protamine, were not only able to bind to the endothelial cell surface but also demonstrated the ability to penetrate the BBB [103]. Currently, it is widely accepted that the electrostatic interaction between positively charged moieties on proteins, and the negatively charged apical surface of the endothelial cells of the BBB conferred by the presence of glycocalyx, are responsible for initiating the process of AMT and allowing uptake of the protein across the BBB [104].

Of clinical interest for the delivery of drugs into the brain are the efflux mechanisms of the BBB, in particular the ATP-binding cassette (ABC) transporters which are responsible for the failure of most therapeutics to enter the brain [105]. There are 49 members of the ABC transporter superfamily, of which many are efflux transporters [9]. P-glycoprotein (permeability glycoprotein), also known as ATP-binding cassette sub-family B member 1 (ABCB1), has been found to be one of the prominent ABC efflux transporters present on the luminal membrane of brain capillary endothelial cells [106]. P-glycoprotein, also known as multidrug resistance protein 1, was originally identified by its ability to grant resistance to multiple drugs in cancer cells by extruding these pharmaceuticals from the cell [107]. Subsequent research on P-glycoprotein found that a staggering range of drugs, can act as substrates for the transporter, ranging from anticancer drugs, such as taxanes and immunosuppressive agent cyclosporin A, to the cardiac glycoside digoxin and glucocorticoids, such as dexamethasone [108]. It is precisely due to the wide spectrum of possible substrates of P-glycoprotein that this efflux transporter poses a major obstacle for biologically active compounds to accumulate in the brain since most of these compounds are actively extruded by P-glycoprotein back into the bloodstream [10]. Efflux transporters have been an area of focus for research, with much work aimed at blocking these transporters, to allow for increased accumulation of drug in the brain. Kemper et al. reported that administration of paclitaxel with a potent inhibitor of P-glycoprotein in a mouse model, allowed paclitaxel to accumulate in the brain to a significantly higher concentration [109]. The study revealed that targeting the efflux mechanisms of the BBB could provide enhanced drug accumulation in the brain.

**Figure 7:**
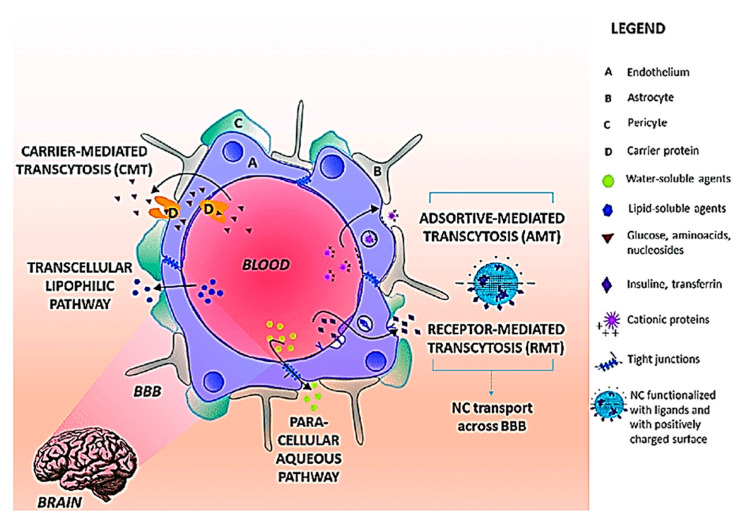
Transport pathways across BBB. The main routes for molecular traffic across the BBB are shown as well as the type of solutes that use each pathway. NC preferential pathways are (i) RMT when the NC surface is functionalized with ligands recognized BBB receptors and (iii) AMT when the NC surface is positively charged. Abbreviations: BBB: Blood Brain Barrier; NC: Nanocarriers; RMT: receptor mediated transcytosis; AMT: adsorptive-mediated transcytosis. Reproduced from [111], with permission from Elsevier, 2018.

While the BBB poses a major hurdle in the delivery of drugs into the brain, it also allows for the opportunity to develop novel approaches to utilize intrinsic endogenous barrier components, such as the influx and efflux mechanisms to allow drugs to enter the brain, instead of “brute-force” techniques like mechanical disruption to physically circumvent the BBB [110,111] Figure 7.

## 4. Nanoparticle-Based CNS Theranostics

**Figure 8:**
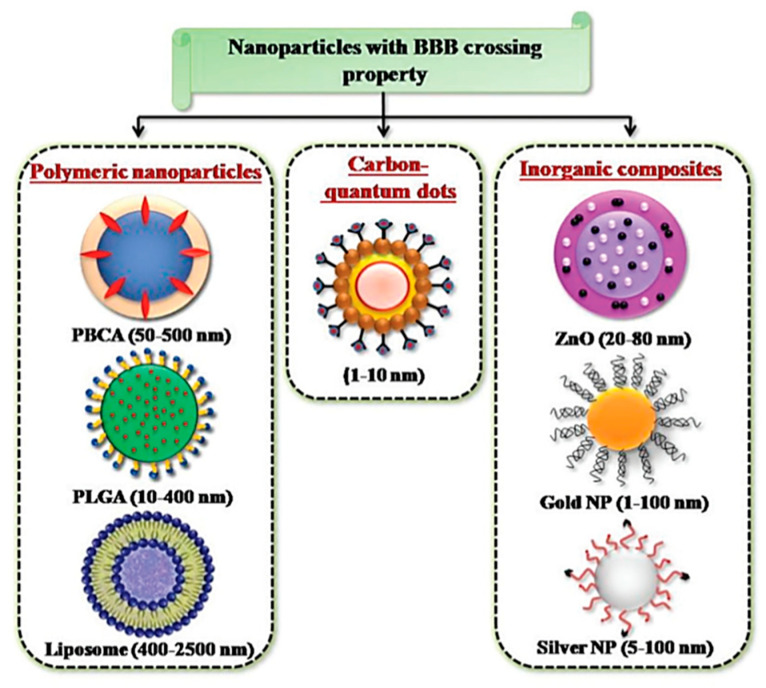
NPs developed for targeted drug delivery into the brain. Reproduced with permission from [58].

The advent of engineered tunable NPs, with sizes in the magnitude of a billionth of a meter, has opened doors to possible therapeutic applications and proposed as a novel approach, to solving the unaddressed problem of improving the transport of biologically active substances across the BBB [12,112]. NPs, being in the scale of 1 and 100 nm, possess peculiar physico-chemical properties with the capacity for modification [14]. NPs are therefore good candidates as drug carriers, due to their small size and consequently large surface area to volume ratio, which would allow the drugs carried to be closer to the surface of the NPs, leading to a faster rate of drug release and higher bioavailability [113]. Furthermore, taking advantage of various biological reactions, such as the receptor–ligand interaction and the antibody-antigen interaction, NPs are able to conjugate with biospecific molecules in a process called functionalization, which allow for targeted delivery of NPs in vivo [114]. The large surface area to volume ratio of NPs, permits multivalent functionalization which drastically increases the binding affinity of conjugated ligand to the desired target receptor [115]. NPs come in various forms and are divided into three primary groups: inorganic nanomaterials (metals, metal oxides, quantum dots and ceramic), organic nanomaterials (liposomes and polymer), and carbon-based nanomaterials [116]. The common types of NPs used in nanomedicine are summarized in Figure 8.

A high functionalization capacity with biological stability, various possible routes of administration and feasibility of adsorbing and distributing hydrophilic and hydrophobic molecules, are what make NPs attractive for medical application [117]. It is noted that systemic administration of non-modified NPs tends to result in non-specific interactions between the NPs and serum proteins, and this adsorption of opsonins on the surface forms a macrostructure called a ‘corona’ which has been found to be readily taken up and cleared by macrophages in the reticuloendothelial system (RES) located primarily in the liver and spleen [118]. To overcome this problem, NPs are typically coated with hydrophilic polymers or surfactants, of which the use of amphipathic polyethylene glycols has been extensively studied [119,120].

As alluded to previously, the BBB is arguably one of the biggest obstacles in the transport of drugs to the brain. Due to their modifiable properties, potential targeting capabilities and prospect of being able to cross the BBB, NPs have emerged as a strong candidate in the treatment of brain disorders [121]. With regard to the ability to cross the BBB, almost all non-modified nanomaterials are BBB impermeable but with a few exceptions [92]. Non-modified silica and gold NPs have been demonstrated to reach brain parenchyma and accumulate through mechanisms that are still unknown [122,123]. One mechanism to improve the uptake of NPs into the brain is via AMT. In essence, functionalizing NPs such that their surfaces bear positive charges will allow these NPs to interact electrostatically with the negatively charged endothelial cells of the BBB, thereby facilitating uptake via AMT. Various methods have been proposed to improve penetration through AMT; for example, Jin et al. demonstrated a marked increase in delivery of solid lipid nanoparticles (SLN) functionalized with cationic cholesteryl hydrochloride and phosphatidyl-ethanolamine [124].

RMT has also been exploited to improve drug delivery using NPs. The technique revolves around functionalizing NPs with ligands of various receptors present on the BBB. Apolipoprotein E (apoE) is a peptide found on the surface of endogenous very-low density lipoprotein (VLDL) and high-density lipoprotein (HDL) which are carriers of cholesterol and other lipids in the plasma and to the CNS [125]. Various groups have shown success in improving NP delivery into the brain via RMT, by taking advantage of the apoE and low-density lipoprotein receptor (LDLR) (its corresponding receptor). Laskowitz et al. synthesized apoE-mimetic peptides expressed as a tandem dimer on liposomes, and noted efficient uptake by the rat brain [126]. The transferrin receptor is another extensively studied receptor for the delivery of NPs across the BBB. Transferrin receptors are present on the apical membrane of the endothelial cells of the BBB, and function to regulate iron uptake via transferrin (which is the plasma protein carrier of iron), whereby binding of transferrin to the transferrin receptors lead to uptake via RMT [127]. Various groups have taken advantage of the high binding affinity of transferrin and its receptor on the BBB, functionalizing NPs with transferrin and have reported successful BBB targeting and brain penetration in vivo [128,129].

### 4.1. Nanoparticle Applications in the Treatment and Diagnosis of AD

The effectiveness of AD drugs currently used clinically is limited by several physiological factors, namely the low permeability of the BBB and the presence of efflux transporters like *p-glycoprotein* which restricts the bioavailability of administered drugs in the CNS [130]. The potential use of NPs in the treatment of AD stem from the ability to tether biologically active substances to NPs, circumventing the barriers highlighted above. Wilson et al. explored the use of poly (*n*-butyl cyanoacrylate) NPs coated with polysorbate 80 and loaded with AD drug rivastigmine in a rat model [131]. The prepared NPs were administered intravenously, and the rats were sacrificed 1 hr post-injection and organs were harvested. The concentrations of rivastigmine in the respective organs were then measured. The same authors demonstrated a 3.82-fold increase in rivastigmine concentration in the brain when the drug was administered via poly (*n*-butyl cyanoacrylate) NPs coated with polysorbate 80, as compared to the administration of free rivastigmine. They hypothesized that the observed increased uptake of rivastigmine in this situation, was in part due to the coating of the surfactant polysorbate 80, which is consistent with results by Borchard et al. in a study using a bovine model, substantiating polysorbate 80 as a highly efficient agent for delivering drugs across the BBB [132]. A subsequent study by Sun et al., investigated the specific role of polysorbate 80 coating on NPs in their uptake into the brain, with findings highly suggestive that polysorbate 80 interacts with the brain micro-vessel endothelial cells, and the authors proposed that the NPs are then taken up by endocytosis [133]. These studies illuminate the vast potential of NPs as nanocarriers to improve biodistribution of commercially available drug for AD, and shed light on the possibility of further improving uptake via functionalization of NPs with surfactants like polysorbate 80.

As previously established, the aetiology of AD while not fully elucidated, can be attributed in part to oxidative damage of the CNS, with such redox reactions catalyzed by transition metals, including copper and iron. Various studies exploring oxidative stress in AD patients and metabolism of iron have shown an elevation of iron levels in AD brains [134]. Liu et al. conjugated iron chelators to NPs and reported a high propensity of these NPs to cross the BBB and high capacity for iron removal from neural tissue [135]. This is a significant finding given the fact that for chelation agents targeting neural tissue to be effective, the chelator must be lipophilic enough to cross the BBB and also capable of leaving the brain, following the formation of the metal ion complex. However, lipophilic chelators often are unable to externalize out of the brain, following formation of the coordination complex with the metal ion due to a change in lipophilicity [135]. The ability for iron chelators to be conjugated to NPs circumvent these problems, since the chemical properties of the conjugated active substance will no longer matter, in the delivery of these agents into or out of the brain.

Various other substrates have been studied to combat oxidative stress from reactive oxygen species (ROS) in AD. Quercetin is a flavonoid compound found in abundance in various food items such as kales, apples, rep grapes and broccoli, and is commonly used in botanical medicine and traditional Chinese medicine owing to its rich antioxidant properties [136]. A study by Kim et al. identified quercetin as a compound capable of inhibiting A*β* fibril formation [137], and given the potential neuroprotective and antioxidant properties of quercetin, it has been widely studied in the context of AD. A study by another group investigated the neuroprotective and antioxidant functions of Quercetin using modified silica NPs as nanocarriers, and showed that the NPs were capable of releasing their drug loads under disorders of Cu (II)-induced oxidative stress in in vitro studies of neuronal and glial cultures [138]. Dhawan et al. studied the effects of Quercetin encapsulated by SLNs in an in vivo rat model [139]. The research team chronically exposed rats to aluminium chloride to increase free radical generation, and cause neurological signs mimicking progressive neurodegeneration and dementia. Spatial navigation tests and elevated maze paradigms were used to ascertain whether there was marked cognitive improvement, following the administration of Quercetin-loaded SLNs. The investigators noted that the quercetin-loaded SLNs were able to reverse the deleterious neurodegenerative effects of aluminium chloride, and the antioxidant properties of quercetin were observed to be enhanced by formulating it as SLNs, with results being highly suggestive that such a nanocarrier system may have useful applications in slowing AD progression and improving cognitive functions.

There have been other studies that have explored targeting the amyloidogenic pathways in AD pathogenesis to slow the progression of the disease. β casein (βCas) is a whey protein which possesses chaperone-like properties and able to bind to a wide array of partially folded peptides (of particular interest A*β*) to prevent aggregation [140]. Javed et al. coated gold nanoparticles with βCas and administered them intracardially into zebrafish which was induced with neurotoxicity from A*β* injected into the cerebroventricular spaces [141]. The authors reported that the administration of βCas gold NPs mitigated the toxicity of A*β* in zebrafish, and displayed an exceptional capability to rescue the animal from AD-like symptoms, as evidenced by results from the various in vivo and in vitro assays conducted.

**Figure 9:**
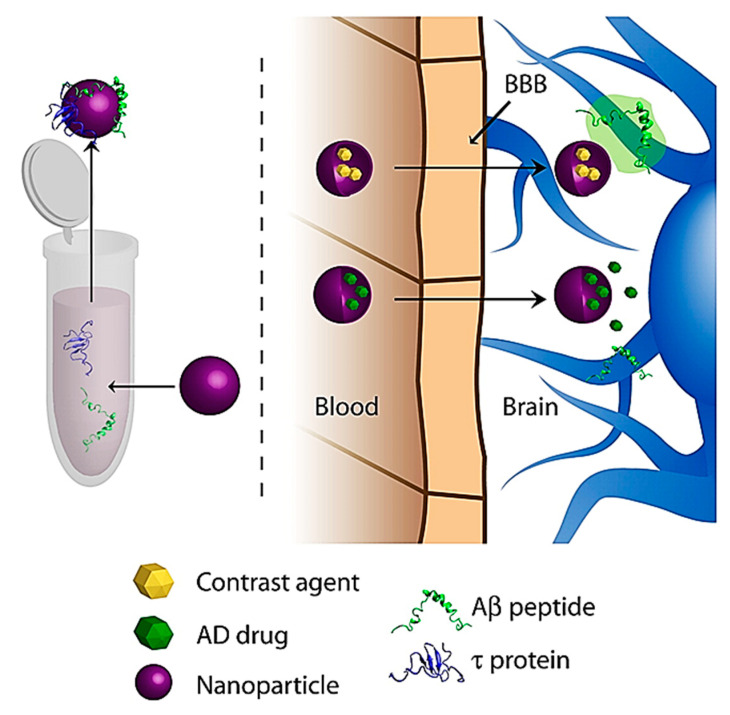
General schematic of NP diagnostic approaches for AD. Reproduced from [142], with permission from Elsevier, 2011.

The use of NPs has also gained interest on the diagnostic front of AD. Most diagnostic efforts aim to carry various contrast agents into the brain, which bind to proteins upregulated in AD, namely tau and A*β* Figure 9 [142]. These contrast agents will then fluoresce or give a signal under various imaging modalities such as magnetic resonance imaging (MRI) [142].

Since its first description in 1959, the fluorescent dye Thioflavin-T (ThT), has been widely used and regarded as the ‘gold standard’ for selective staining and detection of A*β* fibrils both in vivo and in vitro [143]. ThT is a charged compound and if administered in its free form, is unable to pass through the BBB [144]. However, since encapsulation of hydrophilic drugs in NPs has been found to increase drug uptake into the brain [145], it can be extrapolated that encapsulating ThT in NPs may increase its BBB penetration. In the event that ThT can be delivered into the brain via nanocarriers, there should be adequate biodistribution and controlled release for ThT to be a sufficiently effective marker for A*β* and consequently AD. Hartig et al. delivered ThT carried by latex NPs through infusion into the hippocampus of adult mice, and photoconversion of fluorescent ThT was demonstrated on fixed tissues 3 days following the administration of NPs [146]. In this same study*,* electron microscopic analysis demonstrated that there was localization of latex-driven ThT both intracellularly and extracellularly, which suggests that ThT effectively distributes in brain parenchyma, and can be a tool to target both de novo A*β* synthesis intracellularly and A*β* deposits in the extracellular space.

Wadghiri et al. devised a novel imaging method using Aβ1-40 peptide, which is said to have a high binding affinity to A*β* linked to either gadolinium (Gd) or monocrystalline iron oxide nanoparticles (MION), which are magnetic substances used as MR contrast agents [147]. The magnetically labelled Aβ1-40 peptides were co-injected into transgenic mice overexpressing human amyloid precursor protein (APP transgenic mouse model) with mannitol, to increase BBB penetration. Their results showed that systemic administration of Aβ1-40 peptide, chelated to Gd or adsorbed onto MION, and injected with an agent to increase BBB permeability, can be used to detect A*β* plaques by μMRI in the brains of AD-transgenic mice, therefore showing potential for further research as a possible novel imaging method to visualize amyloid plaques.

### 4.2. Nanoparticle Applications in the Treatment and Diagnosis of PD

The progressive neurodegeneration in PD is commonly attributed to the gradual loss of dopaminergic neurons. There has been much research into NP applications for PD, with the focus on restoring physiological dopamine levels as a neuroprotective mechanism. Trapani et al. formulated polymeric chitosan NPs encapsulating dopamine, and conducted in vitro testing on Madin Darby canine kidney II - multi drug resistance gene-1 (MDCKII-MDR1) cell line (an in vitro model of the BBB) to categorize the capacity of the NPs to load and release dopamine [148]. In the same study, in vivo testing on a rat model was also conducted, where rats were acutely treated with the formulated NPs, and microdialysate samples were collected to gauge striatal dopamine concentrations. The same investigators observed an increase in striatal dopamine concentrations and high transportation potential across the BBB. Furthermore, they noted a prompt and pulsatile release profile from the Chitosan NPs. A similar study conducted by Pillay et al. involved the use of dopamine-loaded cellulose acetate phthalate (CAP) NPs, showing a dopamine peak 3 days after the administration, followed by sustained dopamine release [149]. Trapani et al. hypothesized that the prompt and pulsatile nature of the release profile of dopamine from chitosan NPs, was more desirable than the release profile obtained from the CAP nanocarrier used by Pillay et al., citing a lower chance of potential neurotoxicity [148]. Furthermore, chitosan has been found to exhibit a peculiar trait of being able to open the tight junctions of endothelial cells of the BBB and improve the uptake of hydrophilic drugs into the brain, thereby verifying chitosan as a potential nanocarrier of dopamine [150].

Ropinirole (RP) is a non-ergolinic dopaminergic agonist with a high affinity for both D2 and D3 dopamine receptors [151], which has been studied as a potential therapeutic option for PD. Barcia et al. prepared poly (lactic-co-glycolic acid) (PLGA) NPs loaded with RP, and administered the formulation to rats given daily doses of the neurotoxin rotenone to induce neuronal and behavioural changes similar to PD [152]. A series of functional tests were conducted to assess for improvements in cognitive or motor functions of the rats and the results demonstrated that the RP-loaded PLGA NPs were effective in reverting the neurodegeneration in the rotenone-induced PD animal model. A recent study by Dudhipala et al. involved the use of RP-loaded SLNs, and nanostructured lipid carriers (NLCs) formulated as hydrogels for topical administration [153]. In vitro and ex vitro permeation studies conducted confirmed the sustained and prolonged release of RP and significant permeability in the rat model. Pharmacokinetic studies showed that the hydrogel formulations of RP-SLN and RP-NLC resulted in a higher bioavailability of RP than via oral administration, thereby leading the authors to propose topical application of RP-SLNs and RP-NLCs as a possible effective route of administration. The same researchers also confirmed a restoration of biochemical changes in the PD model used, therefore concluding that the NPs used in their study showed potential for further research [153].

Other dopaminergic agonists such as apomorphine have also been explored. Tsai et al. formulated apomorphine carried by SLNs of glyceryl monostearate or polyethylene glycol monostearate, and investigated the biodistribution of the drug in the brain following oral delivery in a PD rat model [154]. The same authors reported that the in vivo drug distribution pattern was highly suggestive that the SLNs successfully targeted apomorphine to the striatum of the brain, and the results also showed a marked improvement in the ability of apomorphine to treat PD. Nerve growth factor (NGF) and other neurotrophic factors have been found to promote growth and survival of various neuronal populations, and some studies have suggested that decreases in NGF levels are observed in various neurodegenerative diseases, such as PD [155]. Kamila et al. devised a novel method involving the brain targeting of NGF using poly (butyl cyanoacrylate) (PBCA) NPs [156]. The same investigators observed that intravenous administration of the NPs carrying NGF, improved cognition and memory and reversed scopolamine-induced amnesia, with a significant reduction in PD symptoms in a PD rat model.

**Figure 10:**
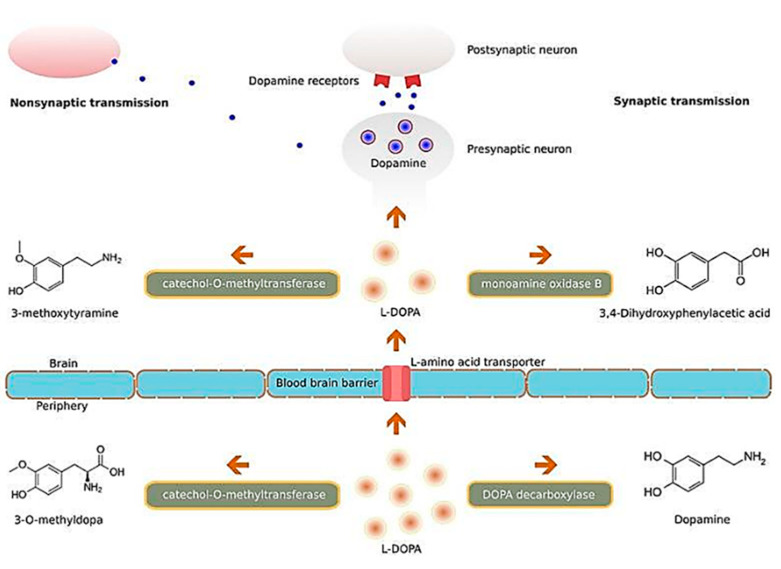
Illustration of the molecular mechanisms of L-DOPA. Abbreviation: L-DOPA: levodopa. Reproduced with permission from [159].

A similar study conducted by Hernando et al. studied the potential clinical significance of another growth factor, glial cell-derived neurotrophic factor (GDNF), in PD [157]. The same group of researchers developed chitosan-coated NLCs for the delivery of GDNF intranasally, and observed from both behavioural studies and immunohistochemistry data that the NP-based formulation was a promising treatment for PD. There have also been studies focusing on nanocarriers encapsulating levodopa such as that by Ngwuluka et al., who developed levodopa-loaded methacrylate copolymer-lipid NPs, and observed that their NP formulation had a high drug loading efficiency of 93% [158]. The authors noted the NPs were capable of sustained release of levodopa and highlighted the potential of their nanocarrier system for possible oral, sustained, and localized drug delivery. Such a nanocarrier system would function by carrying and subsequently releasing levodopa into the brain tissue, which will then be converted into dopamine, and interact with dopamine receptors of neurons Figure 10, so as to ameliorate the imbalance of dopamine secondary to the loss of dopaminergic neurons, as a result of PD [159]. Interestingly, a study by Gao et al. on the intranasal delivery of NPs encapsulating levodopa, reported that surface functionalization of polyethylene glycol-poly lactic acid-co-glycolic acid (PEG-PLGA) NPs, with a lectin derivative of wheat germ agglutinin (WGA), increased the delivery of NPs to the brain by approximately 2-folds [160]. A similar recent study by Arisoy et al. investigated the use of WGA-grafted PLGA NPs, as potential delivery system for intranasal levodopa administration [161]. Significantly, they found that the NPs reached a high target tissue concentration with low dopamine levels in the blood, and showed high clinical efficacy and tolerance in the in vivo rat model used. Their results are highly indicative that such a nanocarrier system could be a potential alternative to the existing levodopa therapy used currently in the clinical setting.

Most research on the diagnostic applications of NPs for PD involve nanobiosensors of dopamine. Shin et al. devised novel silver-molybdenum disulfide (Ag/MoS_2_) NPs for the detection of dopamine, and reported an enhanced electrochemical signal of the synthesized electrochemical biosensor composed of Ag/MoS_2_, showing potential PD applications [162]. Vazquez-Guardado et al. developed an enzyme-free dopamine biosensor system, composed of an active nanostructured plasmonic substrate (NPS), functionalized with oxygen-deficient cerium oxide (CeO_2_) NPs and a passive plasma separator microfluidic chip, and their findings revealed the potential for future complex label-free assays to be developed to target antigens and biomarkers in raw biological fluid [163].

There has also been literature on the potential targeting of α-synuclein for the detection and diagnosis of PD. In one study, gold NP-conjugated aptamers were synthesized, and in vitro testing was conducted to ascertain whether the NP formulation could detect α-synuclein [164]. The authors reported that gold NP-conjugated aptamers, attached on an interdigitated dielectric surface showed high affinity for α-synuclein and therefore suggested that such an NP system may be viable for PD diagnostic applications.

### 4.3. Nanoparticle Applications in the Treatment and Diagnosis of CVDs

Much of the research into the cerebrovascular applications of NPs, have focused largely on the treatment and diagnosis of ischaemic and haemorrhagic stroke. Currently, the clinical gold standard treatment for acute ischaemic stroke is thrombolytic therapy, via an intravenous administration of tissue-type plasminogen activator (tPA), but even this treatment is not optimal and has been shown to have detrimental side effects (such as an increased risk of haemorrhagic transformation), beyond the 3–4.5 h therapeutic window [165].

NPs used in the context of acute ischaemic stroke have involved enhancement of thrombolysis. Zhang et al. prepared liposomes with a cyclic arginylglycylaspartic acid (RGD) motif encapsulating urokinase, a thrombolytic drug [166]. They found that the cyclic RGD functionalization allowed the NPs to bind specifically to activated platelets and improved targeting ability of urokinase for the thrombus. However, it was reported that such a nanocarrier system had a relatively low encapsulation efficiency and loading capacity for urokinase. Hence, this warrants further investigation as to ways for optimizing the drug payload. Despite the shortcomings, the results showed a potential alternative approach to thrombolysis in the clinic. A similar work on NPs carrying thrombolytic agents with high targeting of thrombi has been reported. Juenet et al. synthesized polysaccharide-poly(isobutyl cyanoacrylate) NPs functionalized with fucoidan, a highly sulphated polysaccharide containing L-fucose groups, and studied potential thrombolytic effects [167], as a previous study had shown that fucoidan has a high nanomolar affinity for P-selectin, which is constitutively expressed on activated platelets and endothelial cells, and therefore NP targeting of thrombi can be enhanced by fucoidan surface functionalization [168]. The synthesized fucoidan-conjugated NPs were loaded with recombinant tPA and tested in vivo in a mouse model of venous thrombosis induced by iron chloride [167]. The results showed that their NP formulation carrying recombinant tPA improved thrombolytic efficiency of the drug in the acute phase of thrombosis, substantiating the relevance of targeting P-selectin in treating thrombosis. A recently published study by Liu et al., evaluated the use of CD147 antagonist peptide-9 (AP9)-conjugated NPs directed at the plasma membrane glycoprotein receptor CD147, which has been found to contribute to secondary damage after stroke, by facilitating peripheral leukocyte infiltration and disrupting the BBB [169]. The same authors delivered AP9-conjugated NPs into a mouse model of transient ischaemic stroke, and found a significantly reduced infarct size, with improved functional outcome in the mice tested.

There have also been various studies conducted on NPs for application in haemorrhagic stroke. Jeong et al. studied CeO_2_ NPs and their ROS scavenging abilities in the context of subarachnoid haemorrhage, the most devastating type of stroke, which is known to have a flux of ROS produced in the early progression of the disease [170]. The same investigators synthesized CeO_2_ NPs using aminocaproic acid as a surface modifier, and conducted a randomized trial in an animal model to determine whether free radical scavenging therapy using the synthesized NPs, reduced mortality and improved neurological outcomes. They reported that the NPs displayed a strong capability to protect the brain against subarachnoid haemorrhage, via prominent and versatile ROS scavenging effects, suggesting the potential for future clinical applications, warranting further preclinical testing such as profiling of pharmacokinetics and toxicity effects [170]. You et al. investigated loading NPs with astaxanthin (ATX), a carotenoid with strong antioxidant and inflammatory properties [171]. They synthesized PEG NPs functionalized with transferrin, and loaded with ATX and tested the NPs in vitro on primary cultured neurons. The formulated NPs were found to have favourable biostability, biocompatibility and tissue-specific targeting, demonstrating a significant enhancement in neuronal survival. The authors highlighted that in view of the promising in vitro results, further in vivo research was needed.

There have also been reports of other novel approaches with possible haemorrhagic stroke applications. Bertram et al. developed synthetic platelets based on Arg-Gly-Asp functionalized NPs, and reported that the bleeding time in a rat injury model was halved upon administration of the nanoformulation [172]. In a similar study conducted by Anselmo et al., synthetic platelets developed from polystyrene NPs coated with layers of poly(allylamine hydrochloride) and bovine serum albumin (BSA), and functionalized with collagen-binding peptide, were observed to significantly reduce the bleeding time in a mouse tail resection model [173]. Furthermore, the NPs used by Anselmo et al. appear to mimic the shape, flexibility and surface biology of endogenous platelets, thereby optimizing the accumulation of synthetic platelets at wound sites, and improving the haemostatic functions of endogenous platelets. In another study, silica NPs were reported to be able to increase the activation of the coagulation cascade by adsorbing and stimulating intrinsic pathway coagulation factors [174]. While all the studies were not specifically conducted in a haemorrhagic stroke model, their results showed potential for further exploration as therapeutic agents for haemorrhagic stroke [172,173,174].

Iron oxide NPs have been studied as a potential selective marker for p-selectin and other adhesion molecules, for detection of early neuroinflammation response in acute ischaemic stroke. One study investigated a novel MR molecular imaging method using iron oxide NPs conjugated with P-selectin binding peptide [175]. This study demonstrated that such a nanocarrier system could act as a contrast agent for the in vivo detection of neuroinflammation, 24 hr after an acute bout of stroke induced in a mouse model. Hubert et al. applied a similar nanocarrier system of superparamagnetic iron oxide NPs as contrast agents for MRI analysis in a murine stroke model [176]. The team found that such a NP system, coupled with MRI, could be used to track the involvement of inflammatory cells—for example, phagocytes in the inflammatory onset of acute ischaemic stroke. This study has shed light on the possible utility of NPs in studying immune involvement during neuroinflammation. Lin et al. devised a NP system involving iron oxide NPs conjugated with thrombin-sensitive peptide substrates [177], for surveying the host circulation for thrombi, following intravenous administration. In response to coagulation, there is a release of ligand-encoded reporters in the urine, thereby serving as a potential diagnostic tool in ischaemic stroke caused by thrombi.

### 4.4. Nanoparticle Applications in the Treatment and Diagnosis of TBI

**Figure 11:**
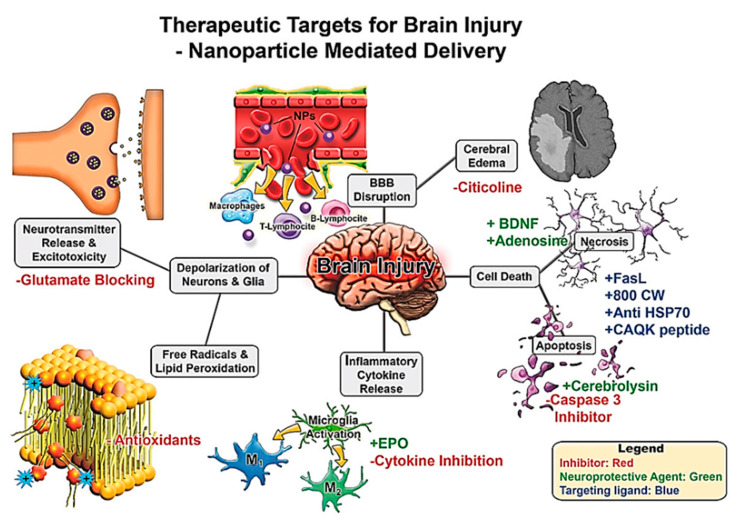
Therapeutic targets for brain injury for nanoparticle mediated drug delivery. Brain injury leads to a cascade of secondary damage events (denoted in grey boxes). These secondary injuries are potential targets for therapeutics where NP delivery may be useful. Potential intervention strategies are highlighted with red/green/blue font where inhibitors/blocking agents are in red, neuroprotective agents via pathway modulation in green, and targeting ligands in blue. Reproduced from [178], with permission from John Wiley and Sons, 2017.

To date, much of the work on NPs in the treatment of TBI involves targeting various underlying pathophysiological pathways such as ischaemia, ROS, neuronal apoptosis, excitotoxicity and neuroinflammation, to discover potential therapeutic targets Figure 11 [178].

Xu et al. developed oxygen reactive polymeric NPs to ameliorate secondary injuries associated with ROS [179]. They copolymerized polyethylene glycol methacrylate with monomer 2-(methylthio)ethyl methacrylate (MEM) and methacrylic acid N-hydroxysuccinimide ester (MNHS) to form the oxygen reactive polymer (ORP). It was observed that the synthesized ORPs had a high reactivity towards ROS, and showed a greater than threefold reduction in hydrogen peroxide levels in vitro, providing evidence of the strong ROS sponge capacity of the ORP. A recent study investigated the use of CeO_2_ NPs and its antioxidant properties [180]. It was found that the CeO_2_ NPs significantly improved the pathological outcomes of mild traumatic brain injury, by reducing neuronal death and oxidative stress, and preserving intracellular signalling and calcium homeostasis. Another recent study explored the efficacy of intravenous immunomodulatory NP treatment for managing neuroinflammation associated with TBI [181]. The same investigators fabricated NPs from carboxylated PLGA and administered the NP formulation intravenously in a mouse TBI model. The anatomic, electrophysiologic, and behavioural data obtained from this study were highly indicative that the immunomodulatory NPs could not only preserve brain matter and function, but also reduce oedema, making them a strong candidate for acute pharmacological intervention of TBI in humans [181].

Yoo et al. investigated core-cross-linked NPs synthesized from polysorbate 80 in a mouse model of TBI [182]. They reported that the NPs were not only able to sequester significant amounts of ROS, but also decreased neuroinflammation and improved recovery and learning. Their results warrant further development and testing as a possible antioxidant and neuroinflammatory therapy for TBI. Khalin et al. conducted a study exploring the delivery of neurotrophic factor to the brain in the treatment of TBI [183]. Brain-derived neurotrophic factor (BDNF) are growth factors responsible for the regulation of neuronal cell growth, proliferation, survival, neuronal plasticity, and long-term memory, and has been suggested as a possible therapeutic option for neurodegeneration [184]. Khalin et al. examined the ability of PLGA NPs coated with surfactant poloxamer 188 to deliver BDNF to the brain, and thereafter studied the neuroprotective effects of BDNF in a mouse TBI model [183]. It was reported in the study that there was an increase in BDNF levels in the CNS, following intravenous administration of the NP formulation (indicating successful BBB penetration), together with a significant enhancement in neurological and cognitive outcomes in the animals induced with TBI. The same study also reported significant neuroprotection in mice with brain trauma, shedding light on the possibility for future clinical applications in the management of TBI in humans.

An interesting approach to control the deleterious injuries associated with TBI, involves the targeted delivery of non-steroidal anti-inflammatory drugs (NSAIDs) to the brain, for managing inflammation due to primary trauma [185]. The investigators developed a nanoprodrug containing Ibu_2_ Tetra ethylene glycol (an ibuprofen derivative) and α-tocopherol. These two components were polymerized together to form NPs which were able to cross the BBB, and provided a large surface area for hydrolytic esterase enzymes to interact and degrade the prodrug, thereby releasing ibuprofen. It was observed that the drug accumulated preferentially in the region of the injured tissue, and therefore there is potential for future translational work, given that its high specificity is also likely to reduce detrimental off-target side effects.

Takahashi et al. has developed a novel approach of conferring neuroprotection in a TBI animal model [186], by designing and synthesizing a nitroxide radical-containing polymer, capable of self-assembly into redox-active nitroxide-containing NPs (RNPs). They observed that the administration of RNPs, with its high ROS scavenging capability, improved cognitive behaviour evaluation, reduced brain contusion volume, and promoted neuroprotective microglia production. Furthermore, the long half-life and high selectivity towards the lesions may have also contributed towards neuroprotection. Interestingly, Takahashi and his research colleagues also explored the use of RNPs to confer neuroprotection in a rat model of cerebral haemorrhage and a mouse model of transient cerebral ischaemia, which similarly yielded very optimistic results of neuroprotection [187,188,189,190]. To date, RNPs display very promising neuroprotective effects that call for further characterization and exploration.

On the diagnostic front of TBI, Wang et al. developed a rapid, highly specific nanosphere system for the detection of neuron-specific enolase (NSE) and S100-*β* protein, which are common TBI biomarkers released into the circulation following brain damage [191]. The nanospheres were designed from gold NPs functionalized with silica, for the sensitive surface-enhanced Raman scattering immunosensor detection of NSE and S100-*β*. The nanosphere system displayed excellent performance for detection of NSE and S100-*β* with a remarkable detection limit, good stability, wide linear range and a good correlation with enzyme-linked immunosorbent assay (ELISA), holding great promise for future clinical diagnostics application. Cruz et al. developed PEG-PLGA NPs encapsulating both near infrared (NIR) fluorophores and perfluorocarbons (PFCs), and conjugated with a cyanide dye 800CW, which were targeted to necrotic cells for the detection and monitoring of TBI and its progression [192]. Following intravenous administration of the NP formulation, the investigators were able to successfully identify the necrotic brain lesions in a mouse model of TBI, via optical imaging and fluorine MRI. Such a targeted NP system has demonstrated the potential for the use of NPs as a non-invasive method for highly specific and sensitive imaging of cerebral lesions of TBI.

## 5. Clinical Status of NPs and Future Perspectives

Despite the many exciting breakthroughs highlighted in this review article, it must be acknowledged that nanomedicine as a field is still in its infancy stage. However, given the great potential for NPs to overcome various hurdles that other therapeutic options cannot, such as the BBB, nanomedicine has been gaining interest over the past decade. Currently, there are over 20 nanomedicines approved for clinical therapeutic or diagnostic use by the FDA or European Medicines Agency (EMA), and more than 75 clinical trials on NPs have been launched over the past few years [193]. It should also be noted that many of the nanodrugs approved for use or in development are directed towards the treatment of cancer [194]. Given the current lack of NPs approved for clinical use or in clinical trials for the treatment of neurological disorders, the breakthroughs highlighted in this review from laboratory-based experiments may one day solve the unmet need for a more efficacious therapeutic regime for neurological disorders. Hence, given the many desirable and unique properties of NPs, more work on their biomedical applications in the treatment of neurological conditions is warranted.

It must be conceded that the development of NP systems also brings with it its own unique set of challenges—a system in the magnitude of a billionth of a meter has biological, technological, and clinical limitations that need to be addressed to achieve consistent clinical impact while ensuring the safety of the patient receiving the nanodrug. There are also some barriers which currently hinder the successful translation of NPs to the bedside. For instance, the heterogeneity of human disease might have a bearing on the eventual success of an NP system—each patient’s pathology may differ slightly and consequently, not every patient will react to the designed NP system the same way. In a similar vein, how a drug reacts in a human may be significantly different from how the drug reacts in an animal model of the same disease and therefore could lead to the failure of clinical translation due to disparities between the experimental model and the human disease [195]. Without clinical trials, potential adverse drug reactions or side effects in humans will be hard to predict, therefore raising potential safety concerns. On an implementation level, it may be difficult for an optimized scale-up synthesis of NPs, which affects the viability of distribution of a given nanodrug. If an optimal high-throughput synthesis of NPs is not viable, pharmaceutical companies may not see the cost-benefit ratio as lucrative and could be less willing to venture into the production of nanodrugs.

Admittedly, there is still much investigation and characterization of the properties of NPs, such as their pharmacodynamics, pharmacokinetics and biodistribution to be done before meaningful clinical translation can occur. Associated problems such as off-target side effects, toxicity, biocompatibility and encapsulation efficiency, are some issues that researchers will have to grapple with. Furthermore, even if an NP system passes clinical trials and is approved for clinical use, there are bound to be other challenges that arise such as the practicality of manufacturing that nanodrug at a large-scale. However, NPs and their highly versatile nature may represent a light at the end of the tunnel, as the ‘revolutionary therapeutic’ of the 21st century. The potential is vast, but more work needs to be done to optimize NPs as a tool to solve the medical challenges of today.

## 6. Conclusions

Neurological disorders are difficult to manage due to the BBB, a physiological interface which bars the entry of many therapeutic molecules into the brain. Increasingly, NPs have been gaining attention as a possible therapeutic option for neurological disorders, given its unique and versatile properties, which can provide solutions to barriers that conventional drugs are unable to overcome. The physical and chemical properties of NPs, such as size and surface charge, have facilitated the transport of therapeutic molecules and drugs across the BBB by these nanocarriers (mediated via AMT and RMT). NPs can be synthesized from a host of different materials and can be conjugated to other materials or molecules. This flexibility in the design of NPs confers them favourable properties such as highly specific tissue targeting, low immunogenicity and high stability in the circulation. In the context of neurological disorders, perhaps the most advantageous property of a nanocarrier system is that the physical and chemical properties of the drug carried by the NP has no bearing on the transport of the drug. This is especially important considering that the BBB excludes most active compounds by chemical and physical properties such as molecular size and hydrophilicity.

Thus, the need for more optimal clinical options for the management of PD has spurred development in the frontiers of nanomedicine, with promising results in the early diagnosis of PD using nanomarkers and the delivery of CNS-targeting therapeutics using nanocarriers [196], which would also be applicable to AD. Similarly, research into NPs as a possible treatment option for TBI has been gaining traction in recent years, since NPs are said to be able to circumvent the biological barriers that hinder the transport of conventional drugs to the brain and encapsulate biologically active substances, reducing off-target side effects while improving site-specific delivery of therapeutics [197]. On-going efforts are also focused on NP-based diagnostics and therapeutics for CVDs—in particular, ischaemic and haemorrhagic stroke.

While the promise that NP-based therapeutics hold is vast, the field of nanomedicine is still relatively new, with much to be learnt and explored. Furthermore, there are multiple challenges that need to be overcome in developing an optimal NP system. As it stands, the lack of clinical trials for NPs directed at treating neurological disorders implies that potential side effects in humans are still largely unknown. Indeed, more research is needed to validate the potential therapeutic applications of NPs and to understand the nanosafety concerns. However, NP-based therapeutics could indeed be the panacea that mankind is actively searching for, in order to overcome the problems associated with conventionally difficult to manage neurological disorders.

## Abbreviations

A*β*	Amyloid beta
ABC	ATP-binding cassette
ABCB1	ATP-binding cassette sub-family B member 1
Ach	Acetylcholine
AChE	Acetylcholinesterase
AD	Alzheimer’s disease
Ag/MoS2	Silver-molybdenum disulfide
AMT	Adsorptive-mediated transcytosis
ApoE	Apolipoprotein E
APP	Amyloid precursor protein
AP-1	Activator protein 1
AP9	CD147 antagonist peptide-9
ATX	Astaxanthin
BBB	Blood-brain barrier
BDNF	Brain-derived neurotrophic factor
βCas	β casein
CAP	Cellulose acetate phthalate
CeO_2_	Cerium oxide
CIs	Cholinesterase inhibitors
CNS	Central nervous system
CVDs	Cerebrovascular diseases
DALYs	Disability-adjusted life-years
ELISA	Enzyme-linked immunosorbent assay
EMA	European Medicines Agency
FDA	Food and Drug Administration
Gd	Gadolinium
GDNF	Glial cell-derived neurotrophic factor
HDL	High-density lipoprotein
ICH	Intracerebral haemorrhage
IL-1	Interleukin-1
IL-6	Interleukin-6
KP	Kynurenine pathway
MDCKII-MDR1	Madin Darby canine kidney II - multi drug resistance gene-1
MEM	2-(methylthio)ethyl methacrylate
MION	Monocrystalline iron oxide nanoparticles
MNHS	Methacrylic acid N-hydroxysuccinimide ester
MRI	Magnetic resonance imaging
NGF	Nerve growth factor
NF-κB	Nuclear factor kappa B
NIR	Near infrared
NLCs	Nanostructured lipid carriers
NMDA	N-methyl-D-aspartate
NO	Nitric oxide
NP	Nanoparticle
NPS	Nanostructured plasmonic substrate
NSAIDs	Non-steroidal anti-inflammatory drugs
NSE	Neuron-specific enolase
ORP	Oxygen reactive polymer
PBCA	Poly (butyl cyanoacrylate)
PD	Parkinson’s disease
PEG-PLGA	Polyethylene glycol-poly lactic acid-co-glycolic acid
PFC	Perfluorocarbon
PGE2	Prostaglandin E2
PLGA	Poly (lactic-co-glycolic acid)
QUIN	Quinolinic acid
RES	Reticuloendothelial system
RGD	Arginylglycylaspartic acid
RMT	Receptor-mediated transcytosis
RNPs	redox-active nitroxide-containing nanoparticles
ROS	Reactive oxygen species
RP	Ropinirole
SLNs	Solid lipid nanoparticles
SNCA	Synuclein alpha gene
SNc	Substantia nigra pars compacta
TBI	Traumatic brain injury
ThT	Thioflavin-T
TNF-α	Tumor necrosis factor alpha
tPA	Tissue-type plasminogen activator
US	United States
VEcad	VE cadherin
VLDL	Very-low density lipoprotein
WGA	Wheat germ agglutinin
WHO	World Health Organization
